# Impaired meningeal lymphatic drainage in *Listeria monocytogenes* infection

**DOI:** 10.3389/fimmu.2024.1382971

**Published:** 2024-04-04

**Authors:** Jian Feng, Yuanzhen Ren, Xilin Wang, Xiaojing Li, Xingguo Zhu, Baokai Zhang, Qi Zhao, Xiaochen Sun, Xinxin Tian, Hongyang Liu, Fan Dong, Xiu-Li Li, Linlin Qi, Bin Wei

**Affiliations:** ^1^ Shanghai Engineering Research Center of Organ Repair, School of Life Sciences, Shanghai University, Shanghai, China; ^2^ Institute of Geriatrics, Affiliated Nantong Hospital of Shanghai University (The Sixth People’s Hospital of Nantong), School of Medicine, Shanghai University, Nantong, China; ^3^ Department of Cardiology, Lanzhou University Second Hospital, Lanzhou, Gansu, China

**Keywords:** meningeal lymphatic vessels, mesenteric lymphatic vessels, lymphatic expansion, lymphatic drainage, *Listeria monocytogenes*

## Abstract

Previous studies have demonstrated an association between lymphatic vessels and diseases caused by bacterial infections. *Listeria monocytogenes* (LM) bacterial infection can affect multiple organs, including the intestine, brain, liver and spleen, which can be fatal. However, the impacts of LM infection on morphological and functional changes of lymphatic vessels remain unexplored. In this study, we found that LM infection not only induces meningeal and mesenteric lymphangiogenesis in mice, but also impairs meningeal lymphatic vessels (MLVs)-mediated macromolecules drainage. Interestingly, we found that the genes associated with lymphatic vessel development and function, such as *Gata2* and *Foxc2*, were downregulated, suggesting that LM infection may affect cellular polarization and valve development. On the other hand, photodynamic ablation of MLVs exacerbated inflammation and bacterial load in the brain of mice with LM infection. Overall, our findings indicate that LM infection induces lymphangiogenesis and may affect cell polarization, cavity formation, and valve development during lymphangiogenesis, ultimately impairing MLVs drainage.

## Introduction

The lymphatic vessel network primarily functions to maintain fluid balance and support the body’s immune function. It is a unidirectional flow system that extends extensively throughout the body, responsible for removing interstitial fluid from capillary filtration and participating in tissue immunosurveillance under normal physiological conditions (1, 2). Meningeal lymphatic vessels (MLVs) are predominantly situated in the outermost layer of the meninges, the dura mater, and are responsible for transporting soluble waste products from the brain to the deep cervical lymph nodes (dCLNs) (3–7). This unique function allows MLVs to significantly contribute to central nervous system (CNS) immunity. It has been reported that neurotropic virus infection in mice leads to encephalitis and results in the expansion of MLVs. And MLVs could transport the virus from CNS to the cervical lymph nodes (CLNs), potentially reducing the severity of the disease (8). Furthermore, lymphatic vessels in the mesentery expand on the intestinal wall near the terminal ileum, leading to surgery in severely affected patients with Crohn’s disease, which suggests that peripheral lymphatic vessels are also closely linked to disease progression (9).

The lymphatic system is essential for maintaining internal balance by controlling the flow of inflammatory mediators, white blood cells, and fluid. Lymphatic expansion and edema in the inflammatory tissue usually indicate decreased function (10–12). The important function of lymphatic vessels is to transport immune cells and inflammatory mediators to initiate immunity (13). The physiological process of lymphatic vessel growth is governed by the signaling pathway involving VEGFR-3/VEGFR-C (14). In addition, the regulation of lymphatic vessel dilation and clearance is influenced by inflammation-related molecules like TNF-α, IL-1β, and histamine (15, 16).

The *Listeria* genus is composed of approximately 20 species of bacteria, among these, *L. monocytogenes* (LM) and *L. ivanovii* are relatively common and dangerous (17). Only LM is known to cause infections in humans (18). In contrast, *L. ivanovii* primarily affects animals, especially ruminants, and is rarely linked to human infections (19–21). Little is known about how LM infection impacts the morphology and function of lymphatic vessels. However, bacteria as pathogens invading the body and lymphatic vessels as important tissue organs exercising immune surveillance functions are closely linked. Some diseases caused by bacterial infections have been reported to share an association with lymphatic vessels. For example, in the advanced stages of bacterial keratitis, the sprouting of corneal lymphatic vessels is also enhanced (22). In an earlier investigation, it was observed that in a meningitis model infected with *S. pneumoniae* in rats, there was a notable decrease in cerebrospinal fluid (CSF) drainage to the CLNs (23).

In this study, we focus on changes in the morphology and function of lymphatic vessel, as well as inflammation and organ lesions caused by LM infection. Interestingly, we found that LM infection induced meningeal and mesenteric lymphangiogenesis and impaired drainage function of MLVs. The decreased mRNA levels of genes associated with lymphatic vessel function indicates that LM infection might hinder the proper cellular organization, cavity formation, and valve development crucial for lymphatic vessel growth. These results may provide new insights into mechanisms of multi-organ injury of LM infection, especially in the CNS and provides some new ideas for the future treatment of LM.

## Materials and methods

### Mice

SPF C57BL/6J adult male mice, aged 8 weeks, were acquired from Changzhou Cavens Model Animal Co., Ltd. They were accommodated in the animal housing facilities of Shanghai University, with 3-5 mice per individually ventilated cage (IVC). These mice were allowed to access water and rat food unrestrictedly, and were kept in a controlled environment at 20°C with 55% humidity, following a 12-hour alternation of light and dark. The mice were intraperitoneally anesthetized before any experimental measures. This study involving animals strictly followed the institutional directives, adhered to the Code of Laboratory Animal Management in Shanghai, and was approved by the Institutional Animal Care and Use Committee at Shanghai University.

### Culture of LM

Pick 1 single colony of LM from Tryptic Soy Broth (TSB) solid medium into 3 ml of TSB or LB culture medium containing streptomycin (50 μg/ml) and incubate overnight at 37 °C, shaker 250 rpm. Take 150 μl of culture medium into fresh 3 ml of TSB culture medium, 37 °C, shaker 250 rpm for 2 hours (h). Remove all the culture medium, centrifuge at 5000 rpm, 4 °C for 5 minutes (min), then resuspend with sterile PBS and adjust the OD_600_ to 1. The bacterial solution was divided into several portions, one portion was doubly diluted, and colony forming units (CFU) were determined after plate-coating and incubation, and the rest was added to an equal volume of cryopreservation solution (40% sterile glycerol), and frozen and stored at -80 °C.

### LM-infected mice

Mice were injected intraperitoneally with combined anesthetics, anesthetized for approximately 1-3 min and placed in a mouse fixator. 150 μl of LM dilution (1.2×10^6^ CFU) was injected into the tail vein of mice. The weight of the mice was recorded and their activity status was observed at the same time every day, and subsequent experimental studies were performed after the mice developed neurological symptoms.

### Quantitative real-time polymerase chain reaction

Following Trizol (Takara) extraction, RNA concentration from tissues was quantified using spectrophotometry. Subsequently, cDNA synthesis was initiated with approximately 1 μg of total RNA and M-MLV reverse transcriptase (Takara). Quantitative real-time PCR (RT-PCR) was conducted in a C1000 thermal cycler (BIO-RAD CFX96) using 2× SYBR green (Takara) and primers that target inflammatory factors and genes correlated with lymphatic vessels. β-Actin was utilized for normalization and relative mRNA expression levels were calculated using 2^-ΔΔCT^ method. Detailed primer sequences are available in **Supplementary Table 1**.

### HE staining

Following perfusion, the intact brains, livers, spleens, and small intestines of the mice were immersed in 4% paraformaldehyde (PFA) for fixation lasting 24 to 48 h. Subsequently, the tissues underwent dehydration using an alcohol gradient, transparency treatment with xylene, and embedding in paraffin. We sliced the paraffin blocks into 3.5 μm sections using a microtome, followed by deparaffinization and rehydration processes. Subsequent steps involved a 5-minute staining with hematoxylin solution, treatment with 1% acid ethanol (1% HCl in 70% ethanol), and rinsing in distilled water. After that, eosin solution was applied for a 3-minute staining period, followed by a dehydration process in an alcohol gradient and clearing in xylene. Finally, observation of the sections was carried out using an Olympus BX53 fluorescence microscope (Tokyo, Japan), with image capture.

### ELISA

Commercial ELISA kits (IL-1β from eBioscience and VEGF-C from westang) were utilized to quantify the levels of IL-1β and VEGF-C in various organs of mice.

### Immunohistochemistry

Brain tissues from mice infected with LM were fixed in 4% PFA at 4 °C for 24 h, then dehydrated in PBS with 30% sucrose, followed by embedding in OCT compound and rapid freezing on dry ice. The frozen OCT-embedded tissue blocks were sliced using a cryostat, then washed with PBS to remove OCT residues. Subsequently, the tissues were moved into a blocking buffer containing 3% bovine serum albumin (BSA), 2% fetal bovine serum (FBS), and 0.5% Triton X-100 before specific antibody staining. For meningeal immunofluorescence staining, after skulls were fixed, the meninges were stripped off the skulls for subsequent antibody staining in the 24-well plate. All images were taken by ZEISS Z1.

### Live mouse imaging

Using a Hamilton syringe fitted with a 33-gauge needle, a consistent flow of 1 μl/min was managed for the delivery of a 5 μl OVA-647 solution (2 mg/ml) into the cerebellar medullary pool. Upon completion of the injection, the needle remained in place for 2 min to avoid any fluid reflux. Subsequently, the dorsal incision was sealed with a wound clip, and the mice were transferred to a warming pad for recuperation post-surgery. The mice are then automatically photographed using the IVIS Lumina Series Live Imager at 30, 60, 120, and 180 min. Then the background fluorescence was uniformly calibrated at the end of filming, keeping the same background for each mouse, and the average fluorescence intensity values were derived. Finally, the fluorescence intensity was statistically analyzed.

### Tissue clearing staining

After anesthesia, mice were perfused three times with PBS containing 20 mg/ml of heparin. The second perfusion needed to be supplemented with 1% PFA and 10% sucrose, while the third with 1% PFA. The small intestines with mesentery were then dissected and immersed in PBS with PFA and sucrose for 2 h. The samples were next washed with PBS, followed by overnight immersion in PBS containing Triton X-100 and DMSO. Gentle shaking was applied throughout all the steps.

These samples were sequentially incubated in several concentrations of methanol (20, 40, 60, 80, 100%) for 2 h each. Subsequently, the intestines and mesentery were treated with a mixture of 30% H_2_O_2_ and 100% methanol (1:10) at 4 °C for 48 h. They were then subjected to sequential incubations in decreasing concentrations of methanol (80, 60, 40, 20%) for 2 h each, and then incubated in PBS (10% DMSO/0.2% Triton X-100) for 2 h. Tissues were blocked at room temperature with PBS (10% DMSO/0.2% Triton X-100/5% normal donkey serum/10 mM EDTA-Na). After overnight incubation, the tissues were moved to the primary antibody in a solution of PBS (10 mg/ml heparin/0.2% Tween-20/5% normal donkey serum/10 mM EDTA-Na) for 72 h. The tissues were washed with PBS (10 mg/ml heparin/0.2% Tween-20/10 mM EDTA-Na) for 12 h at 37 °C, with fluid changes every 2 h. Subsequently, the secondary antibody was added to PBS (10 mg/ml heparin/0.2% Tween-20/5% normal donkey serum/10 mM EDTA-Na) for a 72-hour incubation at room temperature. The tissues were washed at 37 °C with PBS (10 mg/ml heparin/0.2% Tween-20/10 mM EDTA-Na) for 48 h, with solution changes every 8 h.

The small intestines with mesentery were embedded in PBS containing 0.8% agarose, and they were sequentially incubated in several concentrations of methanol (20, 40, 60, 80, 100%) twice for 2 h each, followed by a solution containing dichloromethane and methanol (2:1) twice for 2 h. Subsequently, they received three times of treatment with 100% dichloromethane for 30 min each time, followed by 12-hour incubations for two times in 100% dibenzyl-ether. Finally, the blocks were imaged and photographed using Luxendo MuVi-SPIM-CS light sheet fluorescence microscopy.

### Cytometric bead array

Prepare standards from the original concentration to a 1:256 dilution, then add 50 μl of mixed capture beads. Dilute the test sample 1:1 with diluent, take 50 μl of the diluted supernatant and add it to 50 μl of mixed capture beads. For the negative control, take 50 μl of diluent instead of diluted sample. Then, add 50 μl of PE detection reagent to each assay tube and incubate at room temperature, avoiding light, for 2 h. Add 1 ml of wash buffer, followed by centrifugation at 200 g for 5 min, the supernatant was then carefully removed. Finally, add 500 μl of wash buffer to resuspend the beads in each tube and analyze using a CytoFlex3 flow cytometer.

### Statistics

Each experiment was repeated at least twice to ensure the reliability of the results. Data are presented as mean ± SD or SEM. Student’s *t*-test was used for comparisons between two groups, one-way ANOVA or two-way ANOVA was utilized for multiple comparisons. Statistical analysis were conducted using GraphPad Prism 9.0 software, with statistical significance indicated by P<0.05 (*p < 0.05, **p < 0.01, ***p < 0.001, ****p < 0.0001).

## Results

### LM infection induces meningeal lymphangiogenesis in mice

**Figure 1:**
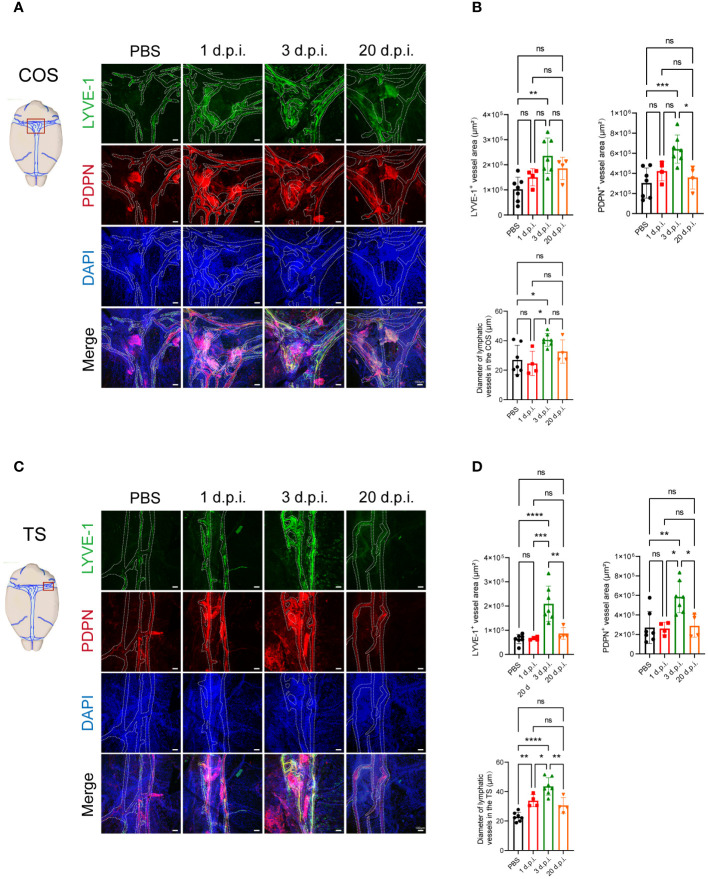
*Listeria monocytogenes* infection induces meningeal lymphangiogenesis in mice. **(A)** Images displaying the staining of LYVE-1^+^ and PDPN^+^ in the confluence of sinuses (COS) were captured at four specific time points following *Listeria monocytogenes* (LM) infection in mice. The delineated fluorescent area was indicated by dashed lines, scale bar 100 μm. **(B)** The fluorescent area of LYVE-1^+^ and PDPN^+^ in COS, as well as the quantification of meningeal lymphatic vessels (MLVs) diameter (n=7, PBS; n=4, 1 d.p.i.; n=7, 3 d.p.i.; n=4, 20 d.p.i.). **(C)** Images displaying the staining of LYVE-1^+^ and PDPN^+^ in the transverse sinuses (TS) were captured at four specific time points following LM infection in mice. The delineated fluorescent area was indicated by dashed lines, scale bar 100 μm. **(D)** The fluorescent area of LYVE-1^+^ and PDPN^+^ in TS, as well as the quantification of MLVs diameter (n=7, PBS; n=4, 1 d.p.i.; n=7, 3 d.p.i.; n=4, 20 d.p.i.). Mean ± SD, one-way ANOVA with Holm-Sidak’s multiple comparisons test **(B, D)**. *p < 0.05, **p < 0.01, ***p < 0.001, and ****p < 0.0001. ns, not significant.

To determine whether LM infection induces meningeal lymphangiogenesis in the CNS, we injected 8-week-old C57BL/6J mice with LM through the tail vein. Subsequently, we conducted immunostaining of the MLVs using antibodies against the classic lymphatic vessel markers LYVE-1 (lymphatic vessel endothelial hyaluronan receptor 1) and PDPN (podoplanin) to investigate the changes (**Figures 1A, C**). We measured the area of LYVE-1^+^ and PDPN^+^ in the confluence of sinuses (COS) and transverse sinuses (TS) in the MLVs. We observed that although there was no significant difference between PBS and 1 day post-LM infection (d.p.i.), the expansions of LYVE-1^+^ and PDPN^+^ vessels in the COS and TS of the meninges were observed at 3 d.p.i., along with increased diameter of vessels (**Figures 1B, D**). Furthermore, we observed a significant decrease in PDPN^+^ vessel area in the COS, as well as in LYVE-1^+^ and PDPN^+^ vessel areas and the lymphatic vessel diameter in the TS at 20 d.p.i., compared to 3 d.p.i. (**Figures 1B, D**). In addition to the COS and TS regions, similar changes were observed in the superior sagittal sinus (SSS) region, such as an increase in fluorescent area at 3 d.p.i. compared to the control group, as well as a significant decrease in PDPN^+^ vessel area and lymphatic vessel diameter at 20 d.p.i. compared to 3 d.p.i. (**Supplementary Figures 1A, B**). This indicates that LM infection in the CNS promotes the expansion of MLVs.

### Acute LM infection causes inflammation and multiple organ damage in mice

Previous reports have shown that LM infection could breach the immunologic barrier in the intestine and spread through the bloodstream to the liver, spleen, and lungs in humans and animals. Moreover, if the infection continues to develop, it can even breach the blood-brain barrier and cause fatal meningitis (24). We next intravenously injected LM or PBS into 8-week-old SPF C57BL/6J mice, and percentages of weight change after infection were taken daily. Mice with acute infection exhibited neurological symptoms, including but not limited to seizures, limb weakness, edema, and ocular discharge, and subsequently died on the third to fourth day (**Supplementary Figure 2A**). Some mice experienced weight loss, lethargy, and reduced activity, but their condition began to improve at 11 d.p.i. until they fully recovered (**Supplementary Figure 2B**). To determine the bacterial load of LM in different organs at indicated time points, we collected organs at 1 d.p.i., 3 d.p.i., 20 d.p.i. and PBS control. The bacterial colony count was recorded after 48 h of incubation. LM was detected in various organs such as the small intestine, brain, liver, spleen, and blood at different stages of illness, and the highest number of LM colonies were detected at 3 d.p.i. (**Supplementary Figures 2C-G**).

Next, we used qPCR to measure the expression levels of inflammatory factors in the brain, meninges, small intestine, liver and spleen. We found increased levels of *Il6, Il10, Il1b, Tnfa* and *Ccl2* in the brain and meninges at 3 d.p.i. (**Supplementary Figures 3A, B**). The transcription levels of *Il1b* was significantly upregulated in the small intestine, liver and spleen after LM infection (**Supplementary Figures 3C-E**). We also measured the protein levels of IL-1β, IL-6, IL-10, IL12P70, MCP-1 and TNF-α in the serum and brain tissue supernatant via cytometric bead array or ELISA kit and found elevated levels of IL-1β, IL-6 and MCP-1, both in the serum and brain (**Supplementary Figures 3F, G**). To investigate whether LM infection leads to organ lesions, we performed HE staining on the tissue sections of brain, small intestine, liver and spleen. We observed that after LM infection, infiltration of inflammatory cells in the brain; small intestine damage, such as destruction to intestinal epithelial cells, loss of crypts, development of a subepithelial space in the villi, villus blunting and fusion; the inflammatory cell infiltration and hepatocellular damage in liver; the morphology of the splenic corpuscle became indistinct (**Supplementary Figures 3H-K**). These observations suggest and are consistent with previous reports that LM infection could lead to inflammation and multiple organ lesions in mice.

### Microglia, astrocytes, endothelial cells and macrophages produce VEGF-C upon LM infection

**Figure 2:**
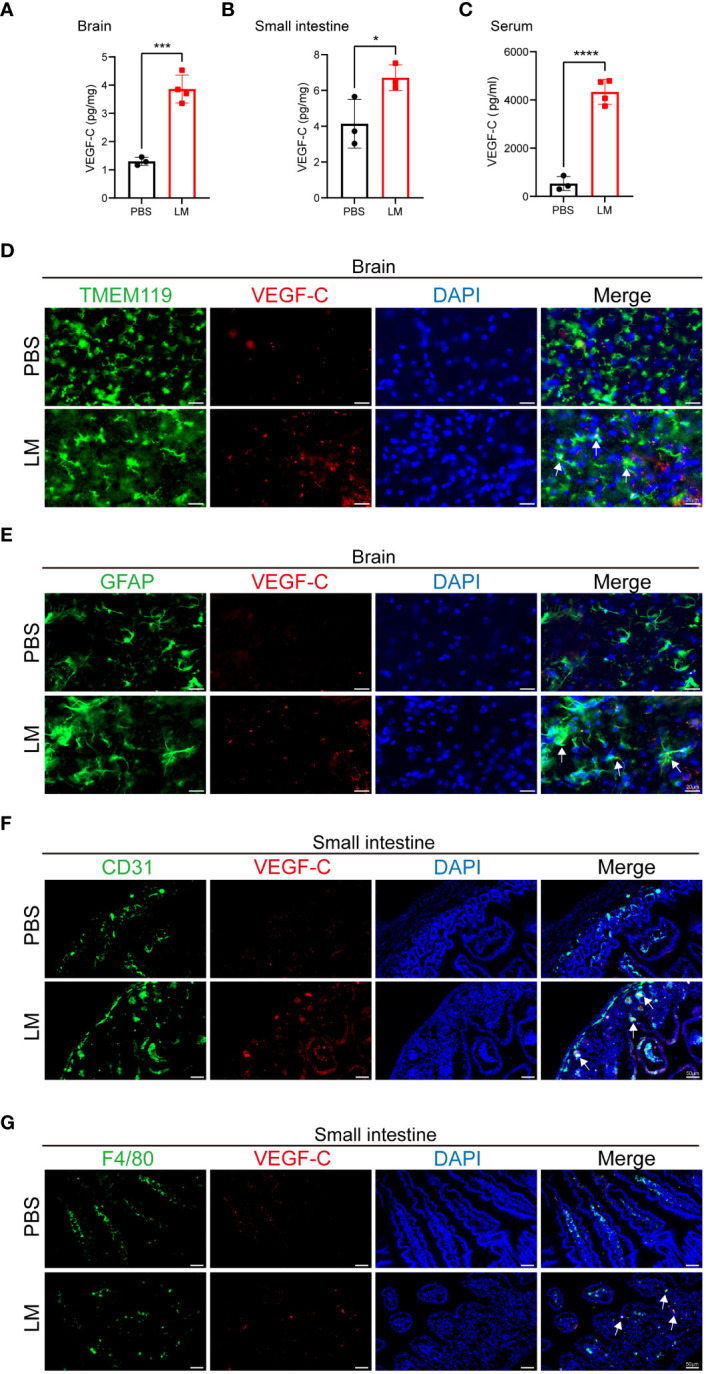
Microglia, astrocyte, endothelial cell and macrophage produce VEGF-C upon LM infection. **(A-C)** VEGF-C levels in brain **(A)**, small intestine **(B)** and serum **(C)** of mice at LM infection 3 d.p.i. compared with the uninfected controls (n=3 PBS; n=4 LM in **(A, C)**, n=3 LM in **(B)**. **(D-G)** Representative images of VEGF-C (red), DAPI (blue) for nuclei, and either: **(D)** TMEM119 (green) and **(E)** GFAP (green) in mice brain sections, or **(F)** CD31 (green) and **(G)** F4/80 (green) in mice small intestine sections at 3 d.p.i. **(D)** The co-localization of TMEM119^+^ microglia and VEGF-C is indicated by arrows. Scale bars in the brain sections, 20 μm. **(E)** The co-localization of GFAP^+^ astrocyte and VEGF-C is indicated by arrows. Scale bars in the brain sections, 20 μm. **(F)** The co-localization of CD31^+^ endothelial cells and VEGF-C is indicated by arrows. Scale bars in the small intestine sections, 50 μm. **(G)** The co-localization of F4/80^+^ macrophage and VEGF-C is indicated by arrows. Scale bars in the small intestine sections, 50 μm. Mean ± SD, a two-tailed unpaired Student’s *t* test **(A-C)**. *p < 0.05, ***p < 0.001 and ****p < 0.0001.

Our study revealed that LM infection causes inflammation of multiple organs, and promotes the expansion of MLVs. VEGF-C plays an important role in lymphatic vessel development, essential for their maturation and functionality (25). Therefore, we detected the levels of VEGF-C in the brain, small intestine and serum of mice at 3 d.p.i. and found that levels of VEGF-C were moderately elevated (**Figures 2A-C**). Microglia and astrocyte also play important roles in inflammation and neurological disorders, as their activation and release of inflammatory mediators can influence disease progression (26). Subsequently, we traced the source of VEGF-C and explored its connection with microglia and astrocyte. Immunofluorescence staining showed that LM-infected microglia and astrocytes produced VEGF-C in the brain (**Figures 2D, E**). We next asked which cell types in the small intestine express VEGF-C and found that VEGF-C was co-localized with some CD31^+^ cells and F4/80^+^ cells (**Figures 2F, G**), indicating that endothelial cells and macrophages in the small intestine could be attributable to the increased expression of VEGF-C following LM infection. These observations suggest that VEGF-C is produced by microglia and astrocytes in the brain, and is also produced by endothelial cells and macrophages in the small intestine during LM infection.

### Impaired MLVs drainage function following LM infection

**Figure 3:**
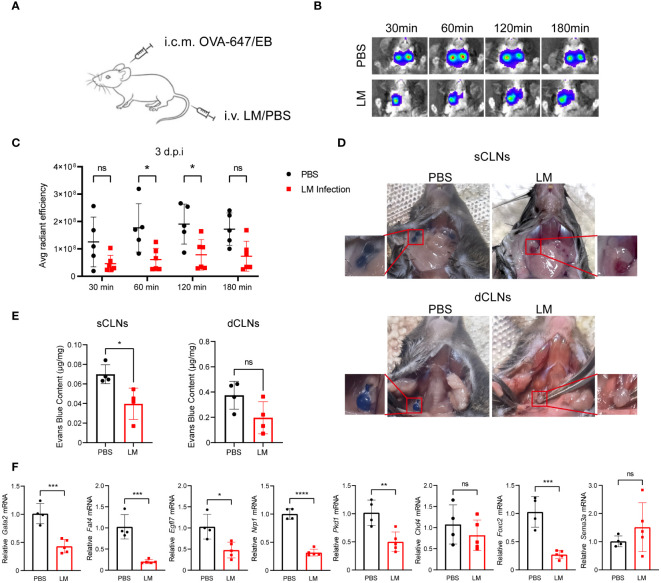
Impaired MLVs drainage function following LM infection. **(A)** C57BL/6J mice were intravenously injected with LM or PBS, and then injected with OVA-647 or Evans Blue (EB) by i.c.m. at 3 d.p.i. **(B)** Representative images of fluorescent intensity of the biopolymer drained to lymph nodes at 647 nm wavelength detected by in vivo imaging at different time points. **(C)** Quantification of OVA-647 fluorescent intensity using the IVIS in vivo imaging system (n=5 PBS; n=6 LM). **(D)** Representative images of sCLNs and dCLNs from mice injected with EB by i.c.m.at 10 min, compared with the uninfected group. **(E)** Quantification of EB intensity in sCLNs and dCLNs of PBS- (n=4) or LM-infected mice (n=4). **(F)** mRNA levels of lymphatic vessel function-related genes in MLVs of mice with or without LM infection (n=4 PBS; n=5 LM). Mean ± SD, a two-tailed Student’s *t*-test **(E, F)** or two-way ANOVA with Holm-Sidak multiple comparisons test **(C)**. *p < 0.05, **p < 0.01, ***p < 0.001, and ****p < 0.0001. ns, not significant.

Our data showed that LM infection promotes MLVs expansion. Hence, we investigated the potential impact of LM infection on the lymphatic vessels’ drainage function. Recent study has proved the role of MLVs in draining cerebrospinal fluid, as well as brain-derived metabolic waste to the CLNs (27). To assess the drainage capacity of MLVs for biological molecules, we administered Alexa Fluor 647-conjugated ovalbumin (OVA-647) or Evans Blue (EB) via intracisterna magna (i.c.m.) injection to both infected and uninfected mice at 3 d.p.i. (**Figure 3A**). We utilized an in vivo imaging system to track fluorescent signals from a ventral view of the mice at different time points following injection in the i.c.m. (**Figure 3B**). After OVA-647 injection, fluorescent signals were detected in the cervical regions. However, these signals appeared notably weaker in the LM-infected mice at 60 and 120 min (**Figure 3C**). Additionally, we injected EB in the i.c.m. of LM-infected and control mice. At 30 min after injection, compared with LM-infected mice, control mice displayed a relatively high accumulation of EB in the superficial CLNs (sCLNs) and dCLNs (**Figures 3D, E**). To identify the mechanism behind impaired MLV function during LM infection, we analyzed gene expression related to lymphatic vessel function in the meninges of LM-infected and control mice. Our findings revealed that the levels of *Gata2*, *Fat4* and *Pkd1*, which are related to cell polarization (28), as well as *Egfl7*, *Nrp1* and *Foxc2*, which are related to valve development (29), were downregulated after infection (**Figure 3F**). Collectively, these findings indicate that the drainage function of dilated MLVs are at least partially impaired in LM-infected mice.

### LM infection induces mesenteric lymphangiectasia in mice

**Figure 4:**
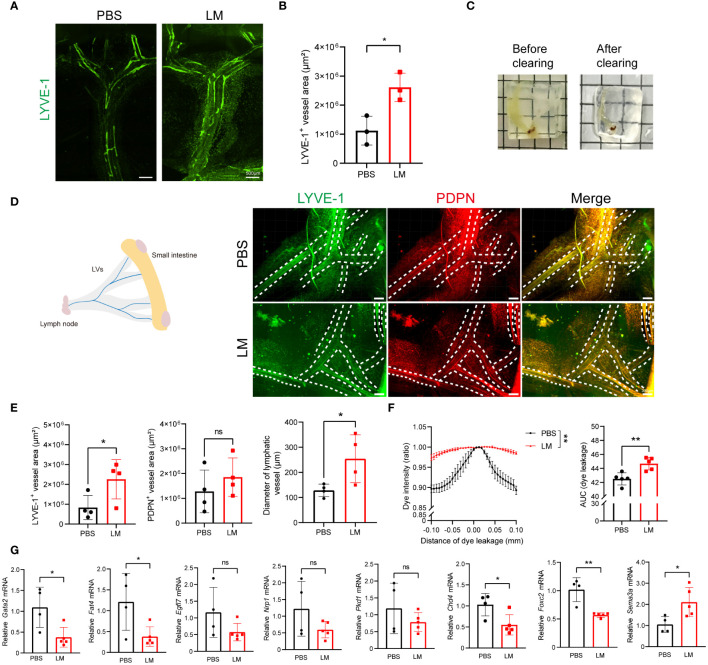
LM infection induces mesenteric lymphangiectasia in mice. **(A, B)** Representative images of LYVE-1 staining **(A)** and measurement **(B)** in mesenteric lymphatic vessels of mice after LM infection, scale bar 500 μm. **(C)** Representative images of the small intestine and mesentery before (left) and after (right) optical clearing. **(D)** Representative images in the optical-clearing-treated mesenteric lymphatic vessels of mice after LM infection, scale bar 300 μm. **(E)** Fluorescent area of Lyve-1^+^ and PDPN^+^, as well as quantification of the diameter of lymphatic vessels are shown (n=4). **(F)** Lift, Graphs show EB dye intensity at the center of the vessel (peak) outward to the surrounding adipose tissue, indicating the quantification of lymph leakage. Right, Area under the curve (AUC) of EB dye intensity plots (n=5). **(G)** mRNA levels of function-related genes in mesenteric lymphatic vessels of mice with or without LM infection (n=4 PBS; n=5 LM). Data are Mean ± SD (Mean ± SEM for panel **D**), two-tailed Student’s *t*-test**(C-E)**. *p < 0.05 and **p < 0.01. ns, not significant.

The mesentery is a critical structure that provides support to the intestine. It contains mesenteric lymphatic vessels, which collect lymph from the intestine to mesenteric lymph nodes. Therefore, we next aimed to determine whether the expansion of mesenteric lymphatic vessels occurs during acute LM infection. For this, we conducted immunostaining of the mesenteric lymphatic vessels using antibodies against LYVE-1 to investigate the changes (**Figure 4A**). We quantified the fluorescent area of LYVE-1^+^ and noted the expansion of the mesenteric lymphatic vessels (**Figure 4B**). To eliminate the potential impact of adjacent adipose tissue, we utilized advanced volume fluorescence imaging through the iDISCO^+^ (immunolabeling-enabled 3D imaging of solvent-cleared organs plus) method. Following the optical clearing process, the intestine and mesentery achieved a high level of transparency (**Figure 4C**). We observed that the fluorescent areas of LYVE-1^+^ and PDPN^+^ in the mesentery were increased after LM infection (**Figures 4D, E**). Additionally, the diameter of the lymphatic vessels was also significantly increased, indicating mesenteric lymphangiogenesis after infection (**Figure 4E**). Meanwhile, we performed EB dye lymphangiography to assess lymphatic function and found a significant expansion of mesenteric lymphatic vessels in LM-infected mice (**Supplementary Figure 4**, **Figure 4F**). For the further study on how LM infection influences the functionality of mesenteric lymphatic vessels in mice, we analyzed the levels of genes associated with lymphatic vessel function and found *Gata2*, *Fat4* and *Chd4*, key regulators of cell polarization, as well as gene *Foxc2* relative to valve development, were downregulated after infection (**Figure 4G**). Together, these findings indicate LM infection could induce mesenteric lymphangiectasia and may affect the function of mesenteric lymphatic vessels.

### MLVs ablation exacerbates inflammation and bacterial load in the brain of mice with LM infection

**Figure 5:**
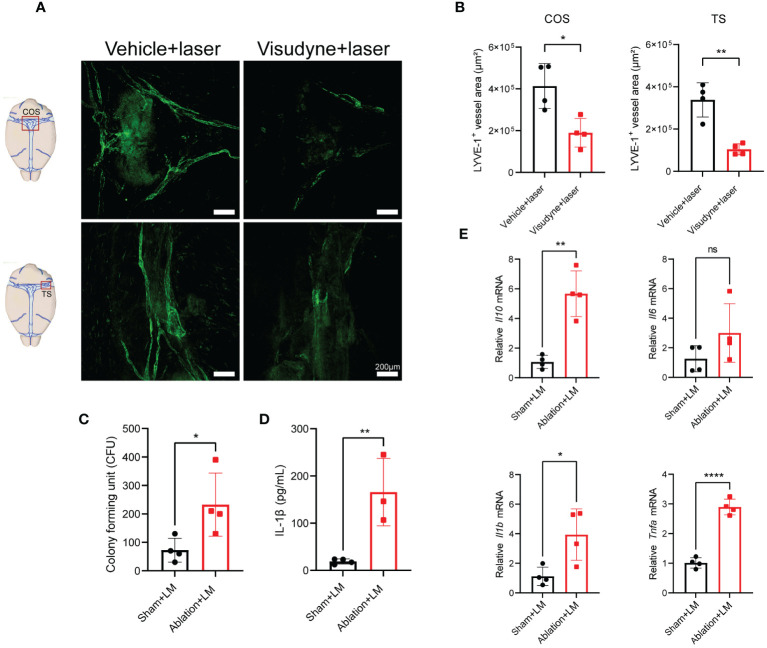
MLVs ablation exacerbates inflammation and bacterial load in the brain of mice with LM infection. **(A, B)** Representative images of LYVE-1 staining in the COS and TS of meninges is presented in **(A)**, accompanied by the quantification of fluorescent area in **(B)** following a 1-week treatment in mice (n=4). **(C-E)** Mice were treated with either PBS or Visudyne, followed by laser irradiation. 7 days post-treatment, the mice received intravenous LM injection. **(C)** The count of LM bacterial colonies in the brain (n=4). **(D)** Measurement of IL-1β levels in the brain at 3 d.p.i. (n= 4 sham; n=3 ablation). **(E)** mRNA analysis of inflammatory factors in the brain at 3 d.p.i. (n=4). Mean ± SD, two-tailed Student’s *t*-test **(B-E)**. *p < 0.05, **p < 0.01, and ****p < 0.0001. ns, not significant.

Subsequently, we explored the potential impact of meningeal lymphatic drainage on bacterial clearance and brain inflammation levels during LM infection. By utilizing a Visudyne-based method (5), we confirmed the effective ablation of MLVs surrounding COS and TS (**Figures 5A, B**). The ablation of MLVs significantly increased the bacterial load in the brain following LM infection (**Figure 5C**). Furthermore, the concentration of IL-1β in the brain after ablation showed significantly increase compared to the sham-treated group, which was infected with LM, but MLVs was not ablated (**Figure 5D**). Additionally, the mRNA expression levels of inflammatory cytokines, including *Il10*, *Il1b*, and *Tnfa*, were upregulated in the brain following lymphatic vessel ablation (**Figure 5E**), which is consistent with the result of bacterial load in the brain. These observations indicate that the ablation of MLVs leads to exacerbation of brain inflammation and bacterial load in LM-infected mice.

## Discussion

LM is commonly found in the environment and often enters the body as contaminant in food. Previous studies have found that LM infection causes the inflammatory response and histopathological lesions in multiple organs, including conjunctivitis, gastroenteritis, lymphadenitis, liver abscesses, splenic abscesses, cholecystitis, peritonitis, pleuropulmonary infections, osteomyelitis, pericarditis, myocarditis, arteritis, and endophthalmitis (30–32). When it breaches the blood-brain barrier, it can cause infections, including life-threatening brain abscesses, meningitis and encephalitis (33). Our study also revealed that LM infection caused inflammation and damage to multiple organs, including the brain, small intestine, liver and spleen. In addition, LM bacteremia can cause leukocytoclastic vasculitis (34). Blood vessels and lymphatic vessels are both components of the circulatory system in mammals. Previous researches have demonstrated that in addition to immune surveillance and metabolite transport, lymphatic vessels are crucial to several diseases, including multiple inflammatory diseases and CNS disorders, such as AD, MS and Glioma (5, 35, 36). However, there is barely relevant researches on LM infection to the morphology and function of lymphatic vessels. Thus, this study aims to elucidate the connection of LM infection with the lymphatic vessel network, and hopefully, will shed light on the new potent strategy against LM infections. Indeed, we found that LM infection caused dilation of lymphatic vessels, especially MLVs, impairing drainage function.

Our previous studies found that neurotropic virus infection can cause expansion and impairment of MLVs (8). It has also been reported that bacterial keratitis can cause sprouting of corneal lymphatic vessels (22), and *Streptococcus pneumoniae* infection leads to decreased drainage of CSF to the cervical lymph nodes (23). Therefore, in this study, the results of LM infection are in accordance with those of several previous researches. Neuroinflammation has been reported to induce lymphangiogenesis near the cribriform plate (37), we speculate that during the occurrence and development of CNS infection, inflammation is associated with the expansion of MLVs in LM-infected mice. Additionally, we observed dysfunction of MLVs and decreased mRNA levels of *Gata2* and *Foxc2* relative to the formation of lymphatic valves after LM infection (38, 39), which suggest that LM infection may lead to impaired formation of lymphatic valves, resulting in dysfunction of MLVs.

Multiple studies have confirmed that VEGF-C/VEGFR-3 is the major molecular driving factor for lymphangiogenesis (40). Previous research in our team has already proven that various cells in the CNS, such as astrocytes and microglia, produce VEGF-C after viral infection, which negatively regulates brain inflammation (41). Other studies have found that VEGF-C and VEGFR-3, abundantly present in human brain tumor macrophages, play a role in regulating inflammation within the tumor microenvironment (42); The VEGF-C/VEGFR-3 can influence ischemic injury progression and modulate astrocyte functions (43). Regarding the specific mechanism of upregulation of VEGF-C expression, previous studies have found that mouse and human macrophages infected with Gram-negative bacteria or under LPS stimulation upregulate the levels of VEGFR-3 and VEGF-C in response to induction of TLR4-MyD88-NF-κB signaling (44). Although LM is a Gram-positive bacterium, there may be a similar mechanism to induce an increase in VEGF-C levels in the brains and intestines of mice, thereby preventing septic shock or cytokine storm.

Although antibiotics can effectively treat and prevent bacterial infections, due to the existence of barrier such as the blood-brain barriers, they cannot effectively treat and intervene in bacterial encephalitis and meningitis caused by a variety of bacteria including LM (45), and new strategies are urgently required to address this infection. Previous studies reported that upregulating VEGF-C in the brain could promote MLV expansion, and enhance the immune response against brain tumors (8). This also raises the question of whether MLVs have a similar ability to clear LM from the CNS. In this study, we found that the ablation of MLVs worsened brain inflammation and bacterial load. Whether it can reduce the inflammatory response and bacterial load in the brain by intervening MLVs remains to be further explored. Therefore, the MLVs can be considered as a potential target for improving bacterial meningitis. Apart from very few tissues such as cartilage and placenta, lymphatic vessels are distributed throughout the body. Infections caused by LM can invade many organs. As the initial site of bacterial invasion and the primary organ of immune defense, the intestines may potentially resist bacterial invasion, eliminate bacteria in the periphery, and reduce mortality by enhancing the immune function of lymphatic vessels.

In conclusion, our study showed that LM infection induced the expansion of meningeal and mesenteric lymphatic vessels and impaired drainage function of MLVs. These results are crucial for understanding the impact of LM infection on lymphatic vessels and developing therapies to enhance lymphatic function. Additionally, it also offers new perspectives on treating LM infection.

## Data availability statement

The raw data supporting the conclusions of this article will be made available by the authors, without undue reservation.

## Ethics statement

The animal study was approved by Ethics Committee of Shanghai University. The study was conducted in accordance with the local legislation and institutional requirements.

## Author contributions

JF: Formal analysis, Investigation, Methodology, Project administration, Writing – original draft, Writing – review & editing. YR: Investigation, Project administration, Writing – review & editing. XW: Investigation, Project administration, Writing – review & editing. XL: Investigation, Project administration, Writing – review & editing. XZ: Investigation, Project administration, Writing – review & editing. BZ: Investigation, Project administration, Writing – review & editing. QZ: Investigation, Project administration, Writing – review & editing. XS: Investigation, Project administration, Writing – review & editing. XT: Investigation, Project administration, Writing – review & editing. HL: Project administration, Writing – review & editing. FD: Project administration, Writing – review & editing. X-LL: Methodology, Project administration, Supervision, Writing – review & editing. LQ: Formal analysis, Investigation, Methodology, Project administration, Validation, Writing – review & editing. BW: Conceptualization, Investigation, Supervision, Writing – review & editing.
