# Supplementary material for: Impaired meningeal lymphatic drainage in Listeria monocytogenes infection
Source: Front Immunol. 2024 Apr 4;15:1382971. doi: 10.3389/fimmu.2024.1382971 (PMC11024298; doi:10.3389/fimmu.2024.1382971)
Supplement: Supplementary file 1 [file DataSheet_1.pdf]

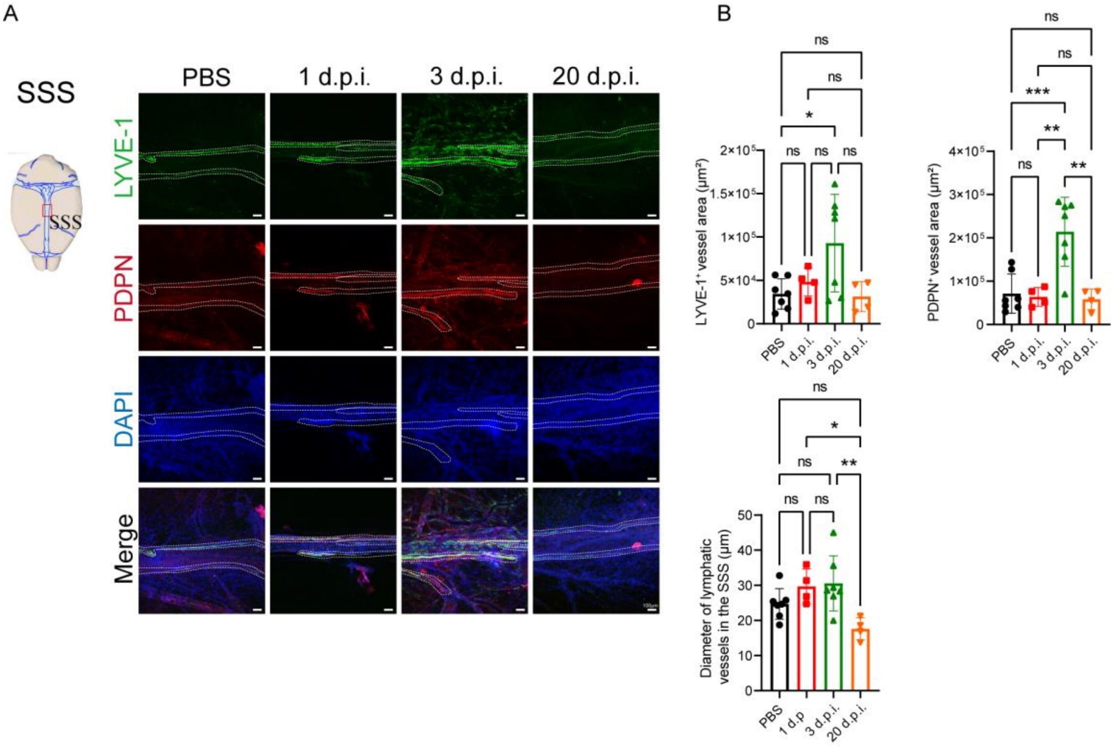

Supplementary Figure 1 LM infection induces the expansion of meningeal lymphatic vessels around the SSS in mice. (A) Representative images of LYVE-1 and PDPN staining in the SSS of MLVs at four time points after LM infection in mice, fluorescent area was marked with dashed lines, scale bar 100 µm. (B) Quantification of Fluorescent area of LYVE-1$^+$ and PDPN$^+$ and the diameter of MLVs (n=7, PBS; n=4, 1 d.p.i.; n=7, 3 d.p.i.; n=4, 20 d.p.i.). All experimental data were repeated at least three times. Mean ± SD, one-way ANOVA with Holm-Sidak's multiple comparisons test (B). ns, not significant, *p < 0.05, **p < 0.01 and ***p < 0.001.

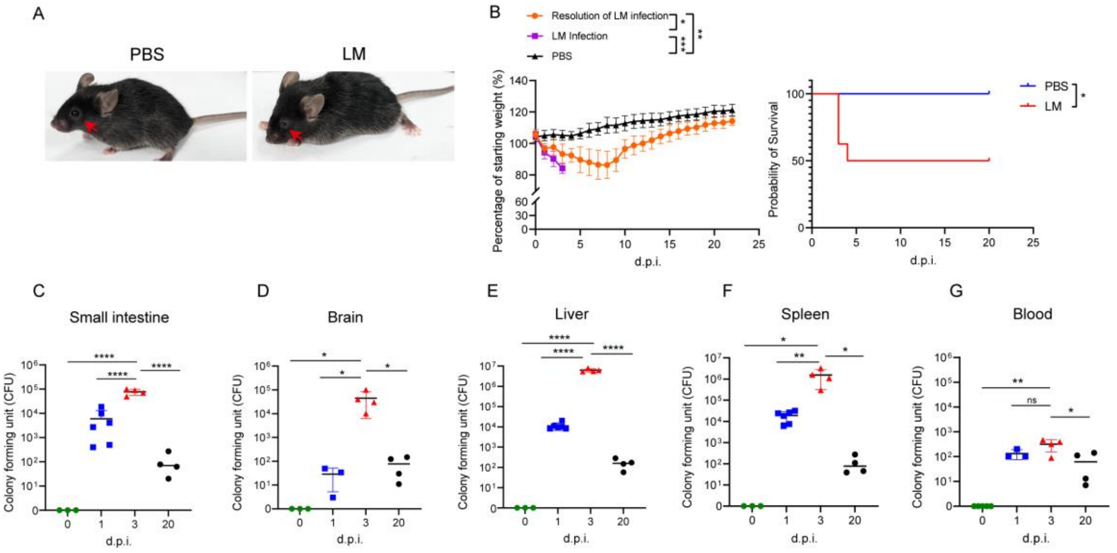

Supplementary Figure 2 Organ distribution of LM after intravenous injection. **(A, B)** 8-week-old SPF-grade male C57BL/6J mice were intravenously infected with 1.2×10^6 CFU of LM or PBS. The percentage change of mice body weight was divided into three groups: PBS group (n=8), LM infection group (n=4), and resolution of LM infection group (n=4). Perform multiple comparisons on the weight at 3 d.p.i. using one-way ANOVA. **B right panel**: Kaplan–Meier survival curve of the different groups. **(C-G)** Bacterial loads in the small intestine (C), brain (D), liver (E), spleen (F) and blood (G) were determined in mice from control group, 1 d.p.i. (early phase), 3 d.p.i. (peak phase), and 20 d.p.i. (resolution phase) (n=3, 4, or 6). All experimental data were repeated at least two times for statistical analysis. Mean ± SD, one-way ANOVA with Holm-Sidak's multiple comparisons test (B-E). ns, not significant, $*p < 0.05$, $**p < 0.01$, $***p < 0.001$, and $****p < 0.0001$.

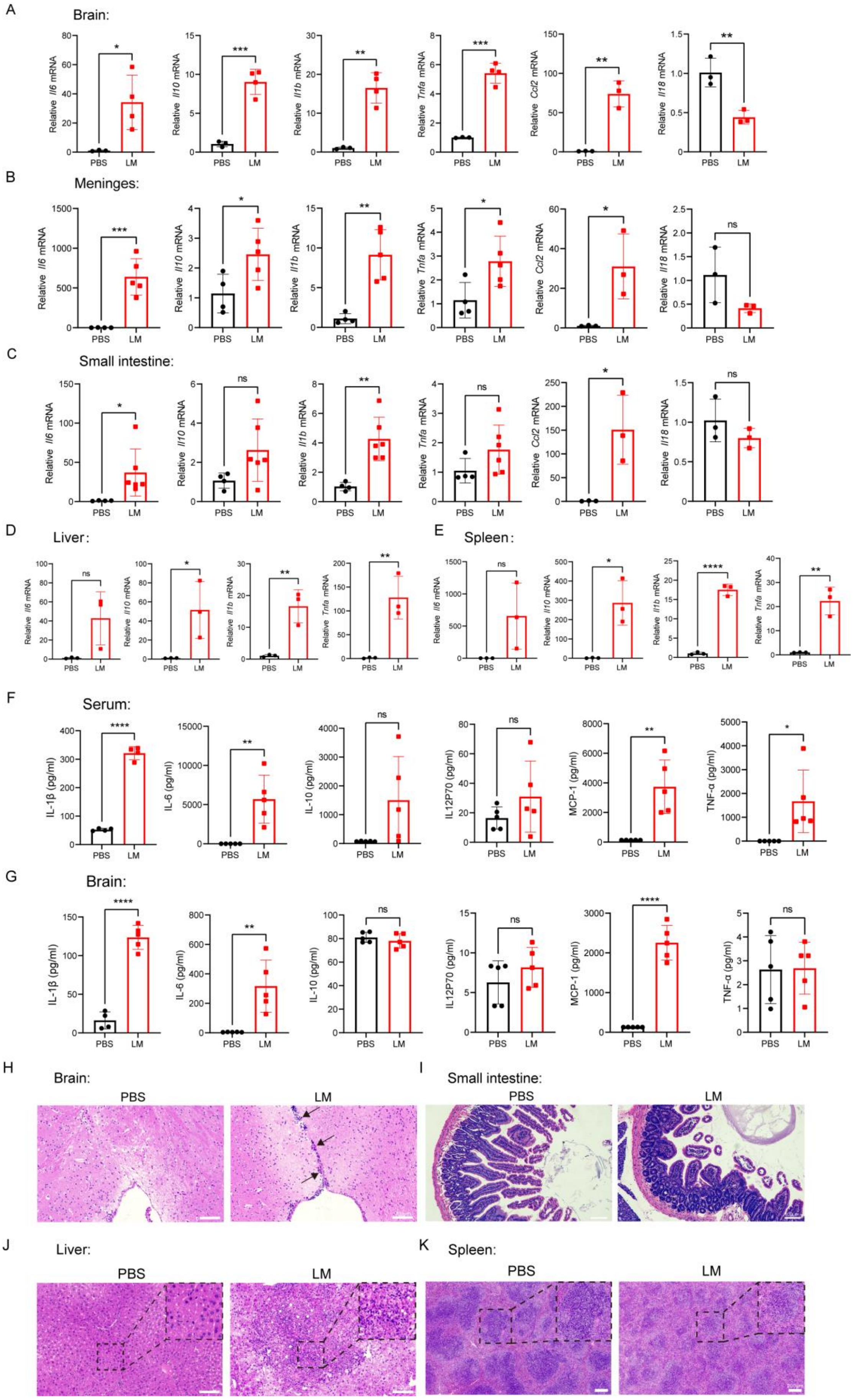
A
Brain:
Relative Il6 mRNA
Relative Il10 mRNA
Relative Il1b mRNA
Relative Tnfa mRNA
Relative Ccl2 mRNA
Relative Il18 mRNA
PBS
LM
B
Meninges:
C
Small intestine:
D
Liver:
E
Spleen:
F
Serum:
IL-1β (pg/ml)
IL-6 (pg/ml)
IL-10 (pg/ml)
IL12P70 (pg/ml)
MCP-1 (pg/ml)
TNF-α (pg/ml)
G
Brain:
H
Brain:
I
Small intestine:
J
Liver:
K
Spleen:
ns
*
**
***
****

Supplementary Figure 3 LM infection causes inflammation and multiple organ damage in mice. **(A-E)** Inflammatory factors related to inflammation in the brain (A) (n=3 PBS; n=3 or 4 LM), meninges (B) (n=3 or 4 PBS; n=3 or 5 LM), small intestine (C) (n=3 or 4 PBS; n=3 or 6 LM), liver (D) (n=3) and spleen (E) (n=3) were quantified by qPCR after LM infection compared to the control group. **(F, G)** Protein levels of inflammation-associated genes in serum (F) and brain (G) were detected by Cytometric Bead Array in LM-infected mice compared to the control group (n=5 PBS; n=5 LM). **(H-K)** Representative images of brain (H), small intestine (I), liver (J) and spleen (K) tissue sections with or without LM infection, showing inflammatory cell infiltration and organ lesions (indicated by black arrows), scale bar, 100 µm (H, I, J), 200 µm (K). All experimental data were repeated at least two times to ensure statistical analysis. Mean ± SD, using a two-tailed Student's *t*-test (A-G). ns, not significant, *p < 0.05, **p < 0.01, ***p < 0.001, and ****p < 0.0001.

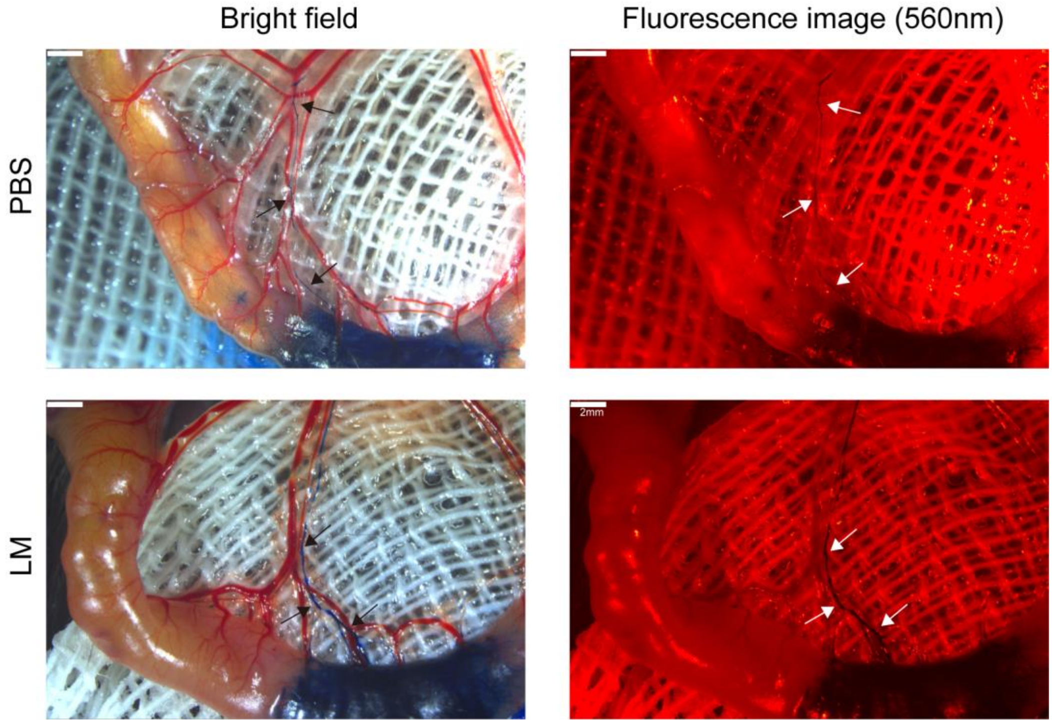

Supplementary Figure 4 LM infection promotes the expansion of mesenteric lymphatic vessels. Representative images of EB dye passage through mesenteric lymphatic vessels and leakage to visceral adipose tissue 10 min after intramucosal dye injection. Representative images of mesenteric lymphatic vessels (Black arrows in left panel, and white arrows in right panel) by brightfield (left pannel) and fluorescence (560 nm, right panel) microscopy. Scale bar, 2mm.

Supplementary Table 1 Primer pairs for qRT-PCR

Gene	Sequence
Il6	F: 5’-TGTATGAACAACGATGATGCACTT-3’
	R: 5’-ACTCTGGCTTTGTCTTTCTTGTTATCT-3’
Il10	F: 5’-CAGGGATCTTAGCTAACGGAAA-3’
	R: 5’-GCTCAGTGAATAAATAGAATGGGAAC-3’
Il1b	F: 5’-CTGGTACATCAGCACCTCAC-3’
	R: 5’-AGAAACAGTCCAGCCCATAC-3’
Tnfa	F: 5’-ACGGCATGGATCTCAAAGAC-3’
	R: 5’-AGATAGCAAATCGGCTGACG-3’
Ccl2	F: 5’-CCGGCTGGAGCATCCACGTGT-3’
	R: 5’-TGGGGTCAGCACAGACCTCTCTCT-3’
Il18	F: 5’-GACTCTTGCGTCAACTTCAAGG-3’
	R: 5’-CAGGCTGTCTTTTGTCAACGA-3’
Gata2	F: 5’-CACCCCGCCGTATTGAATG-3’
	R: 5’-CCTGCGAGTCGAGATGGTTG-3’
Fat4	F: 5’-CAGTGGTGATCCAGGTACGG-3’
	R: 5’-TCATGCGCTGTCACGGAAATA-3’
Egfl7	F: 5’-CTGCTTGTAGCATGGTTTCTAGT-3’
	R: 5’-TACGGCTGGGTCTGTAGACAT-3’
Nrp1	F: 5’-GACAAATGTGGCGGGACCATA-3’
	R: 5’-TGGATTAGCCATTCACACTTCTC-3’
Pkd1	F: 5’-CTAGACCTGTCCCACAACCTA-3’
	R: 5’-GCAAACACGCCTTCTTCTAATGT-3’
Chd4	F: 5’-GCAAAGGTCCCAATGCTCG-3’
	R: 5’-TCGTCGAAGTCAGACTCTACATC-3’
Foxc2	F: 5’-AACCCAACAGCAAACTTTCCC-3’
	R: 5’-GCGTAGCTCGATAGGGCAG-3’
Sema3a	F: 5’-GGCTGGTTCACTGGGATTG-3’
	R: 5’-CCGTTTGCATAGTTTGCTCTGG-3’
β-actin	F: 5’-GGCTGTATTCCCCTCCATCG-3’
	R: 5’-CCAGTTGGTAACAATGCCATGT-3’
